# Fibroblast heterogeneity and FN1-mediated signaling in endometriosis revealed by single-cell and spatial transcriptomics

**DOI:** 10.3389/fimmu.2025.1680849

**Published:** 2025-10-13

**Authors:** Wenwen Shao, Hongmei Ju, Zhikai Xiahou, Sheng Fang, Rugen Yan, Chunyan Li, Yuan Xu, Pingping Cai

**Affiliations:** ^1^ College of First Clinical Medicine, Shandong University of Traditional Chinese Medicine, Jinan, China; ^2^ Department of Gynecology, Affiliated Hospital of Shandong University of Traditional Chinese Medicine, Jinan, China; ^3^ China Institute of Sport and Health Science, Beijing Sport University, Beijing, China; ^4^ Medical School, Kunming University of Science & Technology, Kunming, China; ^5^ School of Integrated Chinese and Western Medicine, Nanjing University of Chinese Medicine, Nanjing, China; ^6^ Department of Gynecology, Shandong Provincial Hospital Affiliated to Shandong First Medical University, Jinan, China; ^7^ Department of Traditional Chinese Medicine, Shandong Provincial Hospital Affiliated to Shandong First Medical University, Jinan, China

**Keywords:** endometriosis, multi-omics, single-cell RNA sequencing, spatial transcriptomics, Fibroblast, FN1

## Abstract

**Background:**

Endometriosis (EM) is a chronic gynecological disorder that affects 5% to 10% of women of childbearing age, often causing pelvic pain and infertility. Fibrosis is a hallmark of EM progression, yet its underlying molecular drivers remain poorly understood. Emerging progress in single-cell and spatial transcriptomic technologies offer new opportunities to unravel the cellular heterogeneity and intercellular interactions driving fibrotic and immune remodeling in EM lesions.

**Methods:**

We performed an integrative multi-omics analysis combining single-cell RNA sequencing (scRNA-seq) and spatial transcriptomics to dissect fibroblast heterogeneity and cell–cell communication networks in EM. ScRNA-seq data from 15 EM patients (GSE213216) were processed to identify transcriptionally distinct fibroblast subpopulations. Functional enrichment (GO, GSEA), stemness estimation (CytoTRACE), and trajectory inference were applied to reveal lineage plasticity. CellChat was used to infer intercellular signaling networks, and spatial transcriptomic data from two ectopic lesions (GSM6690475, GSM6690476) were analyzed to validate the spatial distribution of key ligand–receptor interactions.

**Results:**

We identified 35 cell clusters across EM lesions, with Fibroblast and T/NK cells as dominant populations. Fibroblast were divided into five subtypes, which were associated with extracellular matrix remodeling, immune interactions, and metabolic regulation. Notably, the C2 *CXCR4* ^+^ Fibroblast subpopulation exhibited high proliferative capacity and stemness characteristics, and mediated signaling pathways involved in immune and fibrotic responses through FN1. Spatial transcriptomic analysis confirmed the local enrichment of these Fibroblast in ectopic lesions, where they were associated with regions of active signaling.

**Conclusion:**

This study revealed the transcriptional and spatial heterogeneity of Fibroblast in EM syndrome, and identified the C2 *CXCR4* ^+^ Fibroblast subpopulation as a may represent key driver of fibrosis and immune regulation. Our integrated omics approach provided new mechanistic insights into the pathogenesis of EM and pointed out new targets for therapeutic intervention.

## Graphical Abstract

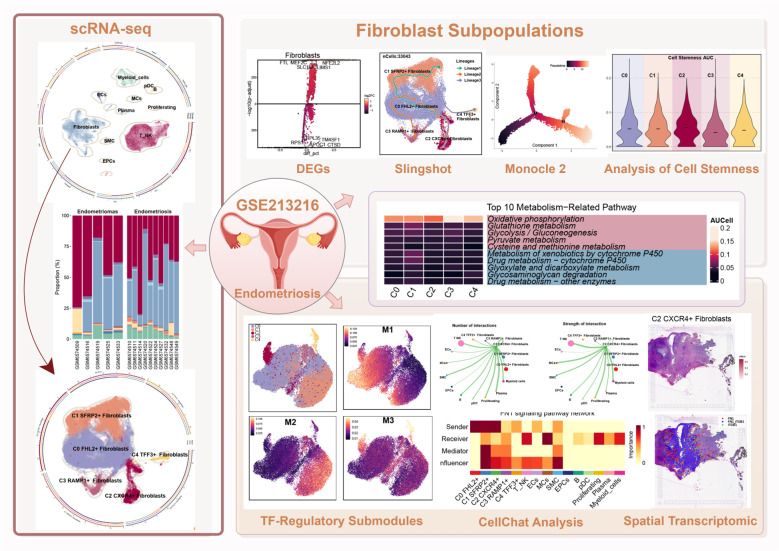

## 1 Introduction

Endometriosis (EM) is a common chronic gynecological disease that affects 5% to 10% of women of childbearing age worldwide (1). The clinical manifestations of the disease are diverse, mainly including dysmenorrhea, chronic pelvic pain and infertility. The disease may involve multiple organ systems, and its symptoms are usually chronic and seriously affect the quality of life of sick women (2, 3). It is also one of the causes of female infertility (4, 5). The exact cause of endometriosis is still unclear, and there is no radical treatment. The current treatment strategy is mainly through hormone suppression and surgical intervention to relieve clinical symptoms, especially pain symptoms. First-line drug therapy, including progesterone and low-dose oral contraceptives, can relieve symptoms in some patients. However, about one-third of patients with endometriosis have little or no therapeutic effect (6). Thus, further investigation into the cellular heterogeneity and spatial organization within endometriotic lesions is essential to uncover the mechanisms underlying EM-associated fibrosis and to identify potential therapeutic targets.

Fibrosis is closely associated with various diseases (7, 8). Vigano et al. have defined EM as a pro-fibrotic state (9, 10). More and more evidence suggests that fibrosis plays a crucial role in the development of endometriosis, suggesting that the treatment of fibrosis may be a promising non-hormone therapy strategy (11). However, endometriosis-associated fibrosis is a complex phenomenon, and its mechanism remains unclear. Platelets, macrophages, ectopic endometrial cells, and sensory nerve fibers have all been implicated in its progression (10, 12).

With the rapid advancement of bioinformatics technologies, significant progress has been made in understanding diverse diseases (13–15), particularly through single-cell RNA sequencing (scRNA-seq) and multi-omics approaches, which provide powerful tools for elucidating disease mechanisms and identifying potential therapeutic targets (16–18). Marcos A. S. Fonseca et al. used scRNA-seq to generate a cellular atlas of the EM microenvironment, revealing dysregulated pro-inflammatory pathways and upregulation of complement proteins in epithelial, stromal, and proximal mesothelial cells (19). Other studies have utilized scRNA-seq to uncover cellular changes in endometriotic lesions, including specific subpopulations of immune-regulatory macrophages, immune-tolerant dendritic cells, and unique vascular changes associated with EM (20). ScRNA-seq has advanced our understanding of cellular heterogeneity and intercellular communication in EM, but lacks spatial context. Spatial transcriptome technology (ST) can directly display gene expression *in situ* on the basis of retaining tissue structure, which makes up for the shortcomings of traditional methods (21, 22). By integrating spatial transcriptomics with single-cell sequencing, we were able to map communication networks within their spatial context. Unlike studies that used scRNA-seq or spatial transcriptomics alone, our combined approach offers a clearer view of how spatially enriched fibroblast subpopulations contribute to fibrosis and immune remodeling in endometriosis.

## 2 Materials and methods

### 2.1 Data acquisition and preprocessing

Single-cell RNA-seq data (GSE213216) were obtained from the GEO database (https://www.ncbi.nlm.nih.gov/geo/). The 10x Genomics datasets were imported and processed as Seurat objects using the Seurat R package (v4.3.0) (23). Doublets were removed using DoubletFinder (v2.0.3) (24). Cells meeting the following quality thresholds were retained: nFeature_RNA ranging from 300 to 5000, nCount_RNA between 500 and 40,000, mitochondrial gene content below 25%, and hemoglobin gene expression less than 5%.

Raw count matrices were log-transformed as log(x + 1). Data normalization was performed using the NormalizeData function in Seurat. A total of 2,000 highly variable genes were selected with FindVariableFeatures, followed by data scaling with ScaleData. Dimensionality reduction was conducted via principal component analysis (PCA), retaining the top 30 principal components (25, 26). Batch effects across samples were corrected using the Harmony package (v0.1.1). Cells were clustered using FindNeighbors and FindClusters (27, 28), and visualized in two dimensions using Uniform Manifold Approximation and Projection (UMAP) (29, 30). Cell cycle phase scores were computed using CellCycleScoring. Cell types were annotated based on canonical marker gene expression and previously published references.

### 2.2 Fibroblast subpopulation analysis

To investigate fibroblast heterogeneity in EM, fibroblast subsets were extracted and re-clustered. The FindAllMarkers function was employed to identify differentially expressed genes (DEGs) among the subpopulations (31). Annotation of fibroblast subtypes was based on their distinct marker profiles. Tissue-specific Ro/e values and cell cycle phases were calculated as described previously (32).

### 2.3 Functional enrichment analysis

DEGs in EM fibroblast subpopulations were evaluated for functional enrichment in Gene Ontology categories with ClusterProfiler (v4.6.2) (33, 34). Additionally, Gene Set Enrichment Analysis (GSEA) (35) utilized KEGG pathway gene sets for the analysis to compare the functional characteristics across different fibroblast subtypes (34, 36, 37). Functional terms were ranked based on statistical significance.

### 2.4 Trajectory and stemness analysis

To infer fibroblast differentiation trajectories, Monocle2 and Slingshot (v2.6.0) were used to carry out pseudotime analysis (38–41). The stemness potential of each subpopulation was quantified using CytoTRACE (21, 42, 43) and subpopulations were ranked accordingly.

### 2.5 Transcription factor and metabolic analysis

Gene regulatory network inference was conducted using the pySCENIC package (v0.10.0) in Python 3.7 (37). Transcription factor (TF) activity in each fibroblast subpopulation was assessed using AUCell, and the top five TFs per subset were visualized using ggplot2. Metabolic pathway activity was evaluated using the scMetabolism R packag (44), and the five most enriched pathways per fibroblast subset were presented as a heatmap.

### 2.6 Cell culture

The ihESC and hEM15A cell lines were utilized in this study. ihESCs were cultured in DMEM/F12 medium supplemented with 10% fetal bovine serum (FBS, Gibco, USA), 1% Penicillin-Streptomycin (PS, Gibco, USA), and 1% GlutaMAX™ (Gibco, USA). hEM15A cells were maintained in DMEM/F12 containing 10% FBS and 1% PS. All cells were incubated at 37 °C in a humidified atmosphere with 5% CO_2_.

### 2.7 siRNA transfection

CXCR4-targeting siRNAs were transfected into ihESCs and hEM15A cells using Lipofectamine™ RNAiMAX (Invitrogen, USA) following the manufacturer’s instructions. A non-targeting siRNA (NC siRNA) served as a negative control. Knockdown efficiency was verified by measuring CXCR4 mRNA expression using quantitative real-time PCR (qRT-PCR).

### 2.8 qRT-PCR analysis

Total RNA was isolated with TRIzol™ reagent (Invitrogen, USA) and subsequently reverse-transcribed into cDNA using the ABScript III RT Master Mix for qPCR with gDNA Remover (ABclonal, China). Quantitative real-time PCR was conducted employing 2× Universal SYBR Green Fast qPCR Mix (ABclonal, China).

### 2.9 Cell proliferation assay

Cells transfected were seeded at 5 × 10³ per well in 96-well plates and allowed to grow for 24 hours. Afterwards, 10 µL of CCK-8 reagent (Beyotime, China) was introduced into each well, and the plates were incubated at 37 °C for 2 hours. Absorbance at 450 nm was recorded using a microplate reader at 24, 48, 72, and 96 hours. Cell proliferation was assessed by plotting growth curves from optical density (OD) measurements, and proliferation differences between groups were analyzed statistically.

### 2.10 Colony formation assay

Transfected cells were plated at a density of 1 × 10³ cells per well in 6-well plates and cultured for two weeks. Afterward, colonies were rinsed with PBS, fixed in 4% paraformaldehyde for 15 minutes, and stained with 0.1% crystal violet for 10 minutes. The colonies were then photographed under a microscope, and their numbers were counted to evaluate cell proliferation ability.

### 2.11 Transwell migration assay

Cell migration capacity was assessed using Transwell chambers (8-μm pore size, Corning). Transfected cells were resuspended in serum-free DMEM/F12 and seeded into the upper chamber, while the lower chamber contained DMEM/F12 with 10% FBS as a chemoattractant. After 24 hours of incubation, non-migrating cells on the upper surface were gently removed. Migrated cells on the lower surface were fixed with 4% paraformaldehyde, stained with 0.1% crystal violet, and counted under a microscope in randomly selected fields.

### 2.12 Intercellular communication analysis

To investigate intercellular communication among different cell types in EM, we applied the CellChat R package (v1.6.1) (45–47). Cell–cell interactions were inferred based on known ligand–receptor pairs using scRNA-seq data (48–50). Both global communication networks and fibroblast subpopulation-specific interactions were quantified and visualized, enabling the identification of key signaling pathways involved in EM pathogenesis.

### 2.13 Spatial transcriptomics analysis of endometriotic lesions

Spatial transcriptomic data from two ectopic lesion sections (GSM6690475 and GSM6690476) were analyzed to investigate cell-type localization and spatial organization. Cell type deconvolution was performed using Robust Cell Type Decomposition (RCTD), with scRNA-seq data serving as the reference. The dominant cell type and proportions at each spatial spot were mapped to spatial coordinates (51).

Spatial feature plots—including total UMI count (nCount Spatial), gene count (nFeature Spatial), mitochondrial gene percentage, stemness AUC, and cell cycle scores—were generated to assess data quality and biological variability. Spatial expression patterns of selected transcription factors and marker genes were visualized across tissue sections.

Spatial domains were identified via unsupervised clustering using the ISCHIA framework. Clustering results were displayed in both tissue sections and UMAP space. Co-occurrence analysis was conducted to assess the spatial proximity and interactions among cell types.

Ligand–receptor interactions within the spatial context were inferred using the stLearn package through both binary and continuous coexpression analyses. Spatially enriched signaling interactions were identified, and their localization patterns were annotated to highlight potential functional niches.

In parallel, the MISTyR framework was applied to construct proximity-aware cell–cell communication networks. Based on multi-scale modeling of tissue microenvironments, MISTyR identified key interacting cell populations and their spatially organized signaling hubs within the lesion tissue.

### 2.14 Statistical analysis

All analyses were performed using R (v4.2.0) and Python (v3.7). Statistical significance between groups was evaluated using the Wilcoxon rank-sum test or Spearman’s correlation as appropriate. Significance levels were indicated as follows: ns, not significant; *P < 0.05; **P < 0.01; ***P < 0.001; ****P < 0.0001.

## 3 Results

### 3.1 Overview of single-cell transcriptomic profiling in endometriotic lesions

We analyzed scRNA-seq data from the publicly available GSE213216 dataset, focusing on 15 samples derived from patients. These included five Endometriomas and ten Endometriosis samples (detailed information for all samples was provided in **Supplementary Table 1**). The overall workflow of this study was illustrated in the graphical abstract.

**Figure 1:**
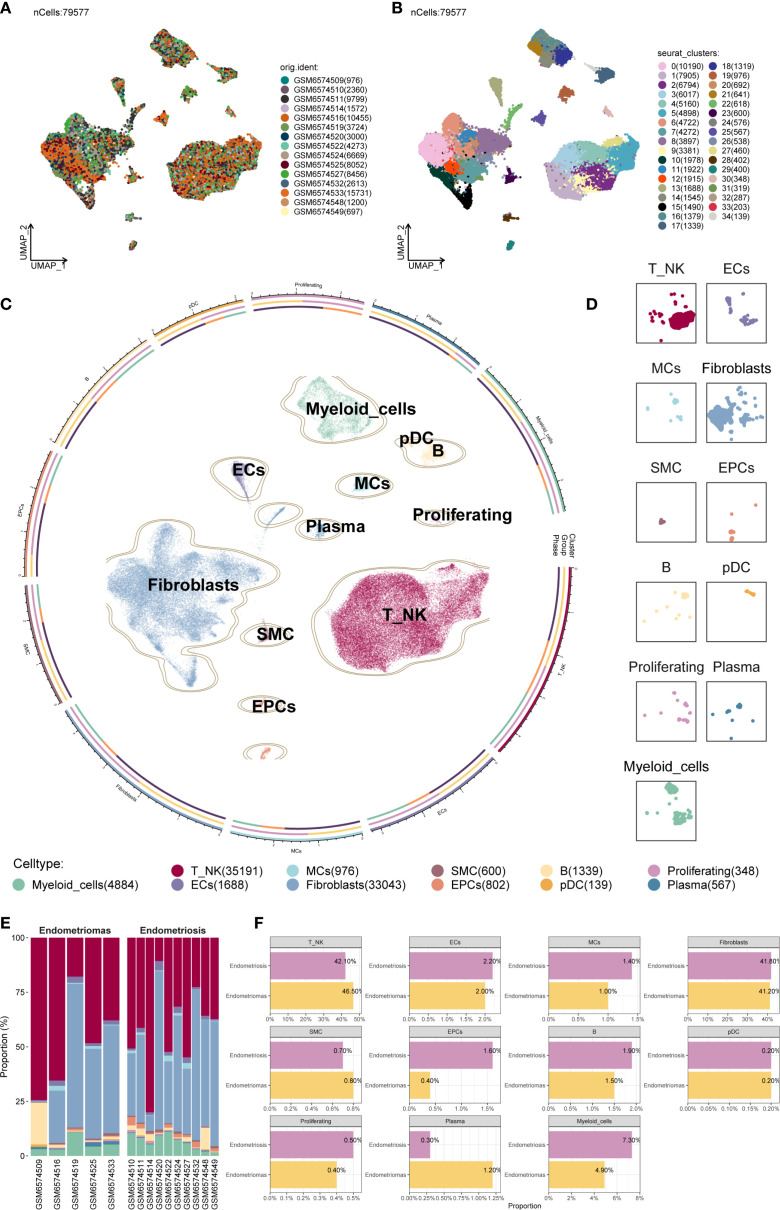
Overview of all cells in EM. **(A)** The UMAP plot showed the distribution of 79,577 high-quality single cells, which were derived from 15 patient samples used in this study. **(B)** The UMAP plot displayed the distribution of Seurat clusters. The high-quality EM single cells were classified into 35 Seurat clusters. **(C)** The circular plot provided a comprehensive view of the distribution of all cell types in EM. **(D)** The UMAP facet plot depicted the specific distribution of each cell type within EM. **(E)** The proportional bar graph demonstrated the distribution of cell and tissue types in each sample. Two tissue types were represented: Endometriomas and Endometriosis. **(F)** The proportional bar graphs presented the proportion of each cell type across different tissue types.

After applying rigorous quality control, a total of 79,577 single cells were retained and subjected to further analysis (**Figure 1A**). These high-quality single cells were clustered into 35 distinct groups using Seurat (**Figure 1B**). Based on the expression of established marker genes, these clusters were further classified into 11 major cell populations. The identified cell types encompassed myeloid cells, plasmacytoid dendritic cells (pDCs), B cells, endothelial cells (ECs), mast cells (MCs), plasma cells, proliferating cells, Fibroblast, smooth muscle cells (SMCs), epithelial cells (EPCs), and a combined group of T and NK cells (T/NK). The circular plot provided an overview of the cellular landscape (**Figure 1C**), and individual UMAP panels illustrated cell-type-specific distributions (**Figure 1D**).

When examining sample-wise contributions, we observed considerable variability in cell type proportions across patients. The bar plot in **Figure 1E** summarized these distributions. Fibroblast and T/NK cells constituted the majority of the cell populations across samples. Of particular interest was patient GSM6574509, who exhibited a pronounced enrichment of B cells alongside a relatively low proportion of Fibroblast, highlighting significant variability between individual samples.

We compared the relative proportions of each cell type between Endometriomas and Endometriosis samples to determine if cellular compositions differed between lesion types (**Figure 1F**). For most cell populations, the distribution was similar between the two groups. As an example, T/NK cells consisted of 42.10% Endometriosis-derived cells and 46.50% from Endometriomas, while Fibroblast showed nearly equal contributions from both sources (41.80% from Endometriosis and 41.20% from Endometriomas), suggesting broadly conserved cellular architecture between lesion types.

### 3.2 DEGs and enrichment analysis of different cell populations in EM

**Figure 2:**
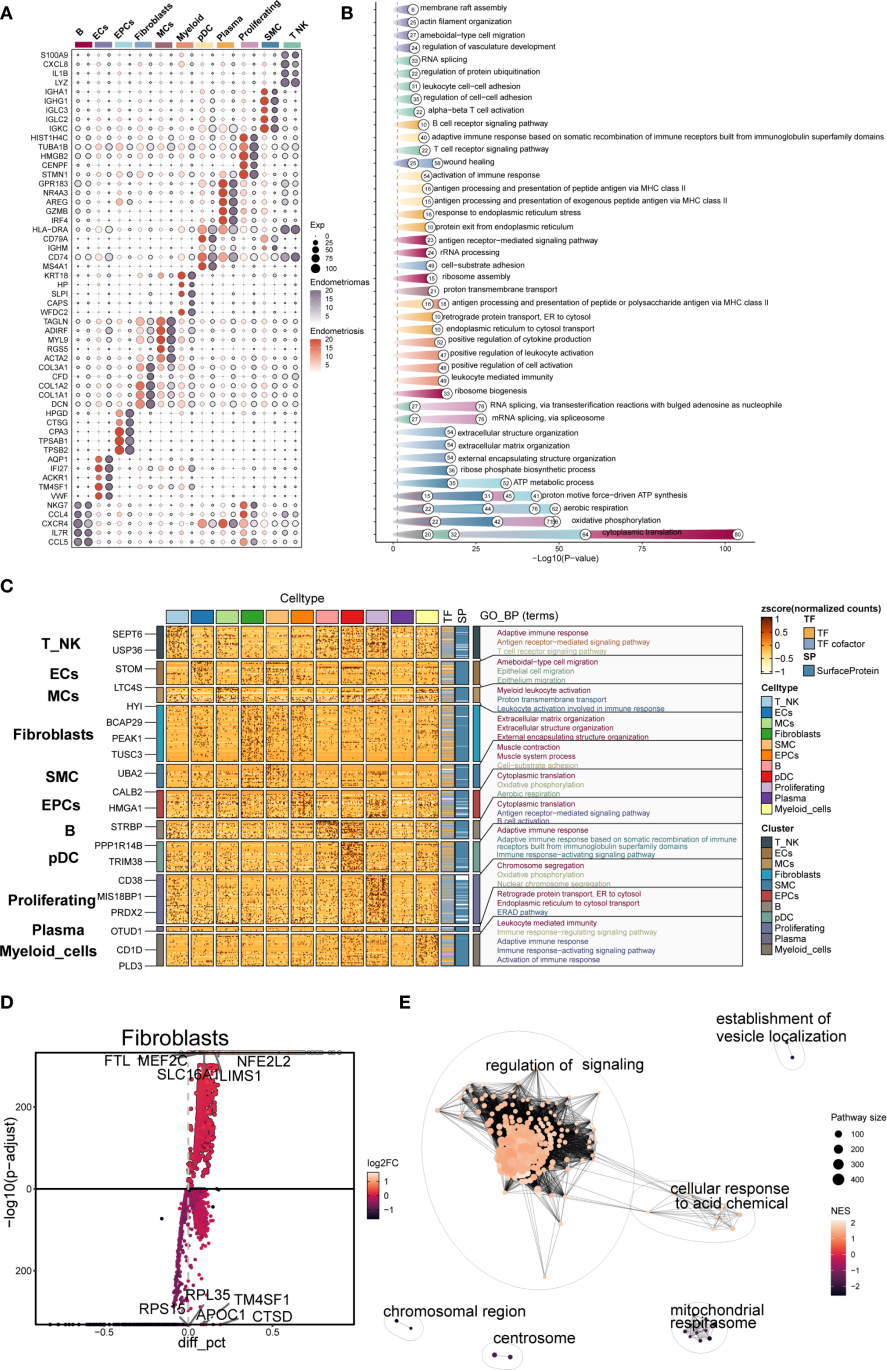
Gene enrichment analysis of different cell types in EM. **(A)** The bubble plot showed the top 5 marker genes for each cell type in EM. **(B, C)** Based on DEGs, the GOBP enrichment analysis results for each cell type in EM were presented. **(D)** The volcano plot displayed the top 5 upregulated and top 5 downregulated genes in the EM Fibroblast subpopulation. **(E)** The enrichment network plot showed pathways enriched in the EM fibroblast subpopulation.

The top five marker genes were determined for each cell subpopulation associated with endometriosis (**Figure 2A**). Fibroblast highly expressed *COL3A1, CFD, COL1A2, COL1A1*, and *DCN*, which were closely associated with extracellular matrix formation and remodeling. Notably, marker genes typically associated with MCs, such as *TAGLN, ADIRF*, and *MYL9*, also showed high expression in Fibroblast, suggesting transcriptional overlap between the two subpopulations.

DEG enrichment analysis was performed for each subpopulation (**Figures 2B, C**). The results indicated that DEGs in the fibroblast subpopulation were enriched in biological processes such as extracellular matrix organization, extracellular structure organization, and external encapsulating structure organization. These processes were critical for EM synthesis, remodeling, and the maintenance of tissue structure, and are fundamental to the interaction between cells and their external environment.

Further analysis of the DEGs in the fibroblast subpopulation (**Figure 2D**) revealed upregulated genes including *FTL, MEF2C, NFE2L2, SLC16A1*, and *LIMS1*, and downregulated genes such as *RPL35, TM4SF1, RPS15, APOC1*, and *CTSD*. Enrichment network analysis (**Figure 2E**) demonstrated that these genes were predominantly involved in pathways related to regulation of signaling, cellular response to acidic chemicals, and vesicle localization, suggesting that Fibroblast may also participate in signal modulation and local metabolic adaptation within the EM microenvironment.

### 3.3 ScRNA-seq revealed the heterogeneity of fibroblast subpopulations in EM

**Figure 3:**
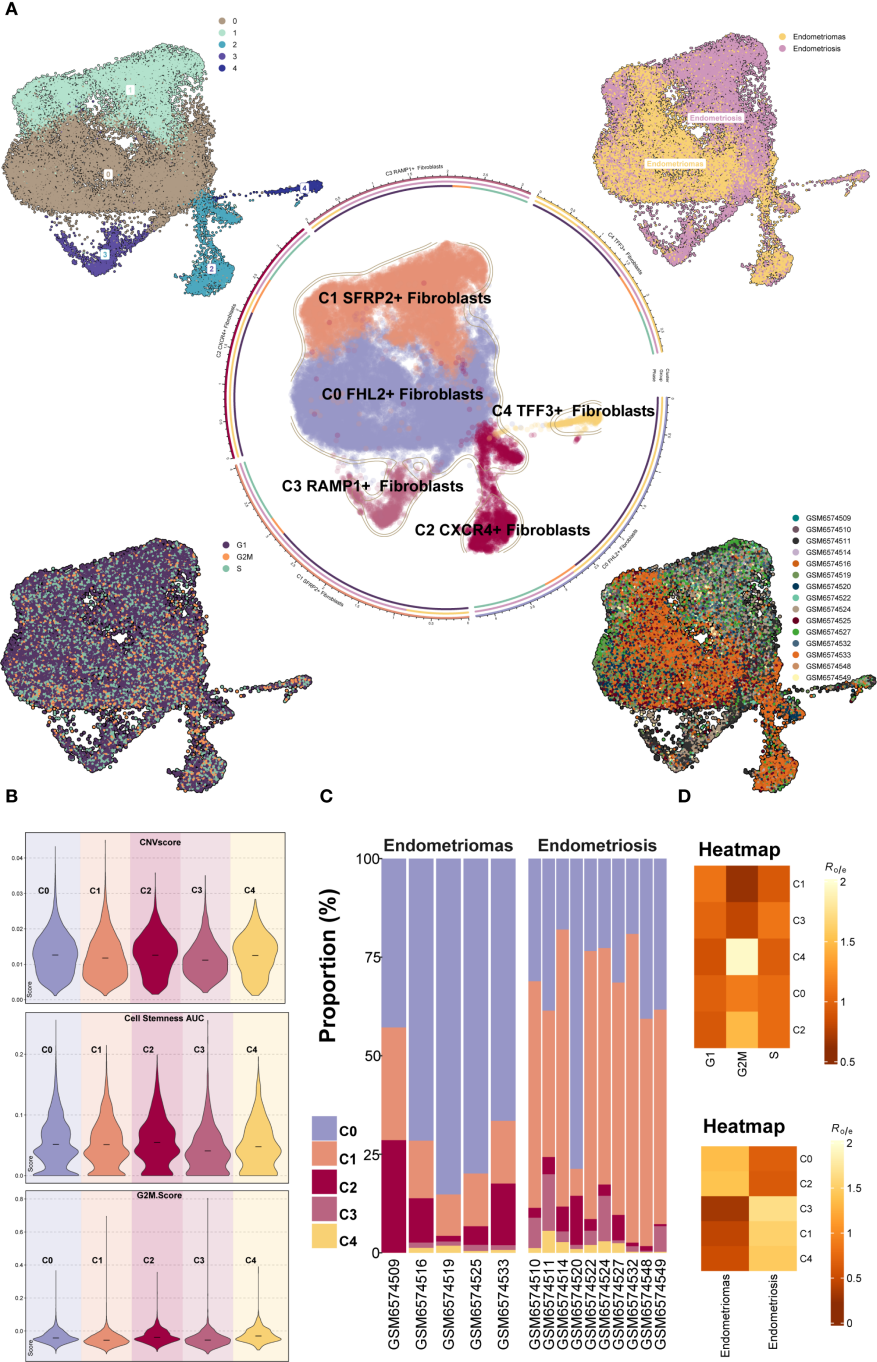
Overview of Fibroblast subpopulations in EM. **(A)** The Fibroblast subpopulations were classified into five Seurat clusters, named based on their marker genes as follows: C0 *FHL2* ^+^ Fibroblast, C1 *SFRP2* ^+^ Fibroblast, C2 *CXCR4* ^+^ Fibroblast, C3 *RAMP1* ^+^ Fibroblast, and C4 *TFF3* ^+^ Fibroblast. The circular plot displayed the distribution of these five Fibroblast subpopulations. The four small UMAP plots around the circle illustrated the Seurat clusters of fibroblast subpopulations, different tissue types, cell cycle stages (G1, G2M, and S), and the distribution across different patient samples. **(B)** The violin plots showed the CNV Score, Cell Stemness Score, and G2M Score for the five EM fibroblast subpopulations. **(C)** The bar graph depicted the tissue type proportions for different EM patient samples and the relative abundance of each fibroblast subpopulation. **(D)** The heatmaps displayed the cell cycle Ro/e values and tissue type Ro/e values for the five EM fibroblast subpopulations.

Further classification of the EM fibroblast subpopulation identified five Seurat clusters, which were named based on their marker genes as follows: C0 *FHL2* ^+^ Fibroblast, C1 *SFRP2* ^+^ Fibroblast, C2 *CXCR4* ^+^ Fibroblast, C3 *RAMP1* ^+^ Fibroblast, and C4 *TFF3* ^+^ Fibroblast (**Figure 3A**). The results indicated that the C1 *SFRP2* ^+^ Fibroblast and C3 *RAMP1* ^+^ Fibroblast subpopulations predominantly originated from the Endometriosis tissue type, while the C0 *FHL2* ^+^ Fibroblast and C2 *CXCR4* ^+^ Fibroblast subpopulations were mostly derived from the Endometriomas tissue type.

To further characterize the heterogeneity among fibroblast subpopulations, the CNV score, cell stemness score, and G2M score were assessed for each of the five EM fibroblast subtypes (**Figure 3B**). The results showed that the C2 *CXCR4* ^+^ Fibroblast had the highest Cell Stemness Score, with relatively high CNV and G2M scores as well. This suggests that this subpopulation may possess strong proliferative capacity and stem cell-like properties. **Figure 3C** showed the proportions of fibroblast subpopulations across patient samples, with C0 *FHL2* ^+^ Fibroblast being the most abundant in Endometriomas tissues.

We further examined the distribution of fibroblast subpopulations across cell cycle stages and tissue types (**Figure 3D**). The results revealed that the C4 *TFF3* ^+^ Fibroblast and C2 *CXCR4* ^+^ Fibroblast subpopulations were more likely to be in the G2M phase of the cell cycle, suggesting a higher proliferative activity. Regarding tissue type preference, the C0 *FHL2* ^+^ Fibroblast and C2 *CXCR4* ^+^ Fibroblast subpopulations were more common in Endometriomas, while the C3 *RAMP1* ^+^ Fibroblast, C1 *SFRP2* ^+^ Fibroblast, and C4 *TFF3* ^+^ Fibroblast subpopulations were predominantly found in Endometriosis. These findings were consistent with the previous analysis.

### 3.4 DEGs and enrichment analysis of fibroblast subpopulations in EM

**Figure 4:**
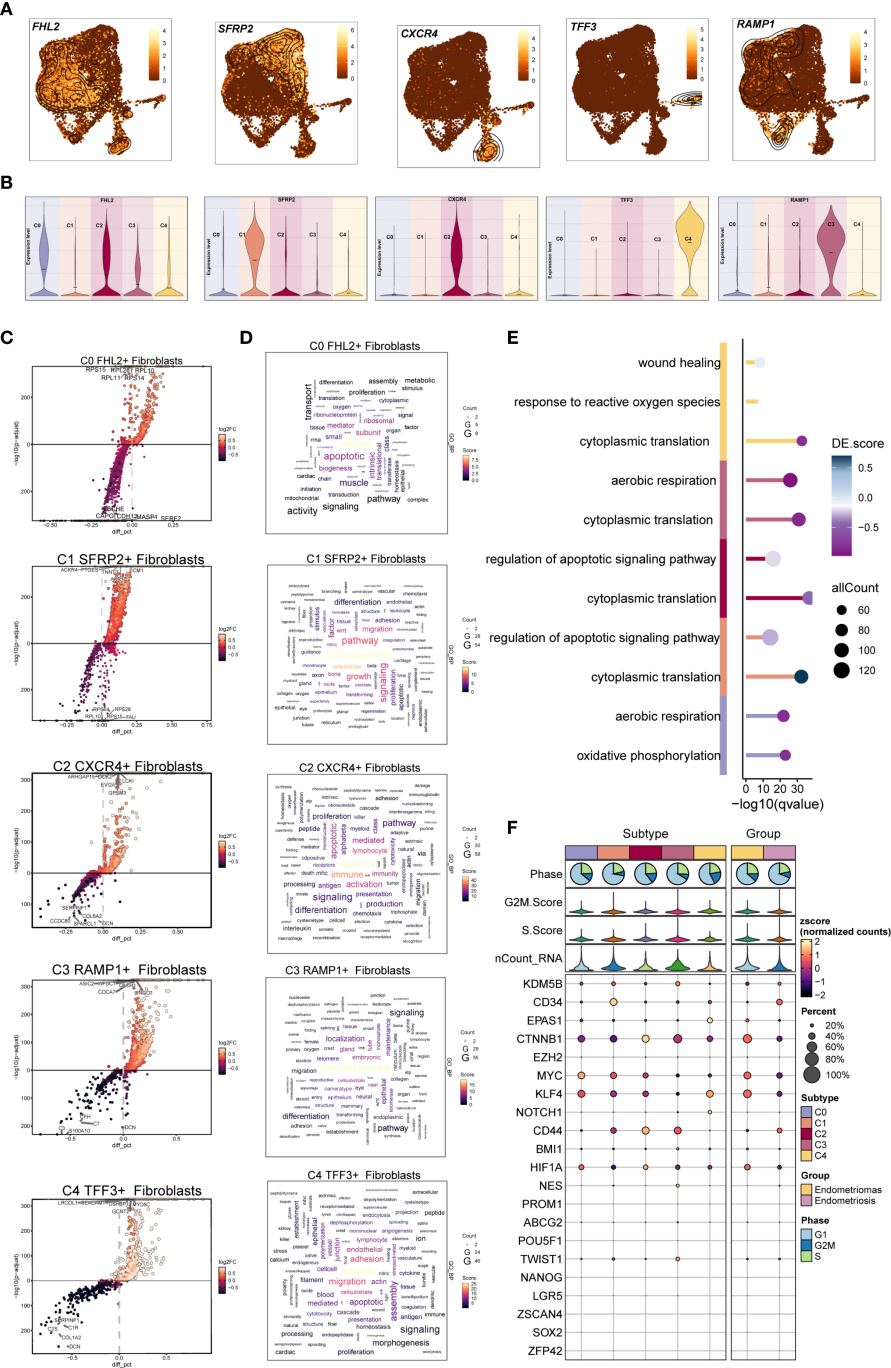
DGEs and enrichment analysis of EM Fibroblast subpopulations. **(A)** The UMAP plots displayed the distribution of the five fibroblast subpopulations marker genes in EM. **(B)** The violin plots illustrated the expression levels of the five marker genes across the EM fibroblast subpopulations. **(C)** The volcano plots showed the top 5 upregulated and top 5 downregulated genes in the five EM fibroblast subpopulations. **(D)** The word clouds represented the enrichment analysis results of the DEGs in the EM fibroblast subpopulations. **(E)** The GO enrichment analysis results for the DEGs of the EM fibroblast subpopulations were presented. **(F)** The bubble plot displayed the stemness genes for each EM fibroblast subpopulation.

To further investigate fibroblast heterogeneity in EM, we analyzed marker genes, DEGs, and their enrichment. The distribution and expression patterns of marker genes for each subpopulation were shown in **Figures 4A, B**. Most subpopulations showed high expression of their respective marker genes. However, *FHL2*, the marker of the C0 subpopulation, was also highly expressed in the C2 subpopulation, suggesting a potential functional link between these two subpopulations.

We then analyzed the upregulated and downregulated genes of each fibroblast subpopulation and performed GO and KEGG enrichment analysis (**Figures 4C–E**). In the C0 *FHL2* ^+^ Fibroblast subpopulation, upregulated genes such as *RPS15, RPL28, RPL10, RPL11, and RPS14* indicated active protein synthesis. These DEGs were enriched in oxidative phosphorylation and aerobic respiration, suggesting elevated energy metabolism. In the C1 *SFRP2* ^+^ Fibroblast subpopulation, the top upregulated genes included *ACKR4, PTGES, TNNT3, ECM1*, and *AKR1C2*. Enrichment analysis showed involvement in morphogenesis, signaling, migration, and regulation of apoptotic signaling pathways. Notably, cytoplasmic translation was a prominent process, implying a potential role of C1 *SFRP2* ^+^ Fibroblast subpopulation in tissue remodeling and cell migration.

The top five upregulated genes in the C2 *CXCR4* ^+^ Fibroblast subpopulation were *ARHGAP15, DOK2, EVI2A, GPSM3*, and *LCK*. Enrichment analysis indicated that the C2 subpopulation was involved in immune responses related to leukocytes and lymphocytes, with a characteristic regulation of apoptotic signaling pathways. Additionally, cytoplasmic translation was notably enriched in this subpopulation, suggesting its potential role in immune response and apoptosis regulation.

In the C3 *RAMP1* ^+^ Fibroblast subpopulation, the top five upregulated genes were *ASIC2, WFDC1, LINGO1, CDCA7* and *DACH1*. Enrichment analysis revealed that C3 was primarily associated with morphogenesis and localization, with biological processes including cytoplasmic translation and aerobic respiration. These results suggest that the C3 subpopulation may play a key role in cellular energy metabolism and morphological remodeling.

For the C4 *TFF3* ^+^ Fibroblast subpopulation, the top five upregulated genes were *LRCOL1, USHBP1, MYO5C, GCNT2*, and *CEACAM1*. Enrichment analysis linked C4 to leukocyte functions, migration, adhesion, and endothelial activity. The response to reactive oxygen species was also one of its prominent biological processes. These findings highlight the important role of the C4 subpopulation in immune response, cell migration, and oxidative stress. .The bubble plot displayed the stemness-associated genes for each EM fibroblast subpopulation, showing that stemness-related genes such as CTNNB1, MYC, and KLF4 were expressed to varying degrees across all fibroblast subpopulations (**Figure 4F**).

### 3.5 Differentiation trajectory of EM fibroblast subpopulations via pseudotime analysis

**Figure 5:**
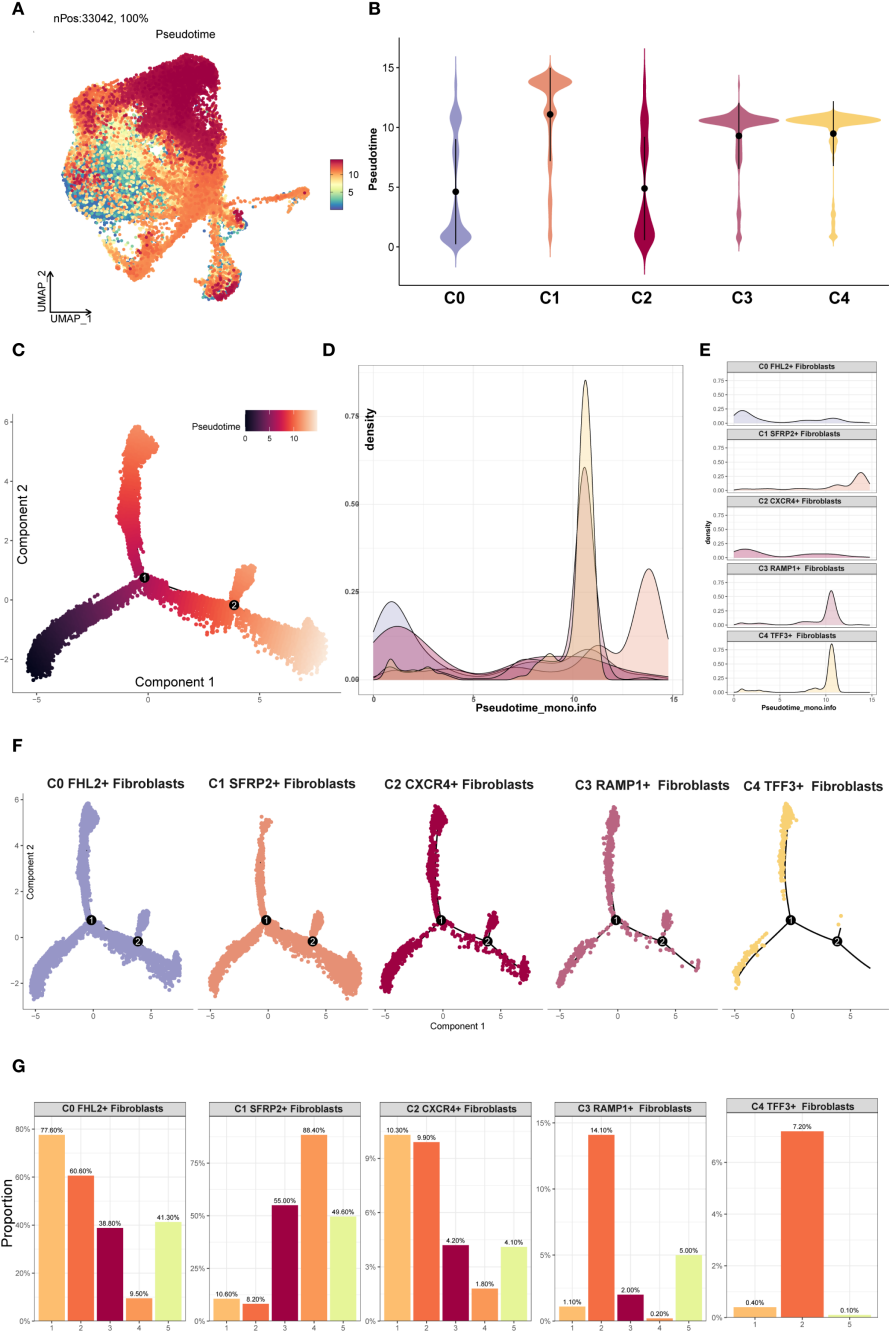
Monocle2 analysis of EM Fibroblast subpopulations. **(A)** The UMAP plot showed the Monocle2 pseudotime results for the EM fibroblast subpopulations. **(B)** The violin plot illustrated the pseudotime trajectories for each EM fibroblast subpopulation. The C2 subpopulation was positioned at the start of the pseudotime trajectory, while the C1 subpopulation was located at the end. **(C)** The pseudotime trajectory plot depicted the progression from the lower left to the upper right. At branch point 1, the trajectory diverged into two paths, with one further splitting at branch point 2 into two distinct trajectories. **(D)** The ridge plot displayed the density changes of each EM fibroblast subpopulation along pseudotime. **(E)** The facet plot provided a detailed view of the density changes of each EM fibroblast subpopulation along pseudotime. **(F)** The facet plot presented the pseudotime trajectories for each EM fibroblast subpopulation. **(G)** The proportional bar graphs showed the specific proportions of each EM fibroblast subpopulation across different pseudotime states.

Differentiation trajectories of EM fibroblast subpopulations were inferred through pseudotime analysis with Monocle 2 and Slingshot. The Monocle 2 results were shown in **Figures 5A, B**. Violin plot analysis indicated that C2 *CXCR4* ^+^ Fibroblast were predominantly located at the early pseudotime stage, whereas C1 *SFRP2* ^+^ Fibroblast were mainly distributed at the terminal end. According to Monocle 2, the pseudotime path originated from the bottom left and bifurcated into two trajectories—one heading upward and the other downward—as it moved toward the top right (**Figure 5C**). The distribution of different EM fibroblast subpopulations along the Monocle 2 Pseudotime trajectory was shown in the facet plot (**Figure 5F**), which revealed that the C3 *RAMP1* ^+^ Fibroblast and C4 *TFF3* ^+^ Fibroblast subpopulations were less concentrated in the lower right branch. Ridge plots were generated to visualize the distribution of each EM fibroblast subpopulation along pseudotime (**Figures 5D, E**). C0 *FHL2* ^+^ and C2 *CXCR4* ^+^ Fibroblast clustered near the beginning of the trajectory, while C1 *SFRP2* ^+^ Fibroblast were mainly found at the end.

The EM fibroblast subpopulations were categorized into five states, and the proportion of each subpopulation in different states was shown in **Figure 5G**. The analysis revealed that the C0 *FHL2* ^+^ Fibroblast subpopulation accounted for 77.60% of state 1, while the C2 *CXCR4* ^+^ Fibroblast subpopulation accounted for 10.3% of state 1. This distribution likely reflects the difference in cell numbers, as the C0 *FHL2* ^+^ Fibroblast subpopulation contained 17,794 cells, whereas the C2 *CXCR4* ^+^ Fibroblast subpopulation contained 2,482 cells.

**Figure 6:**
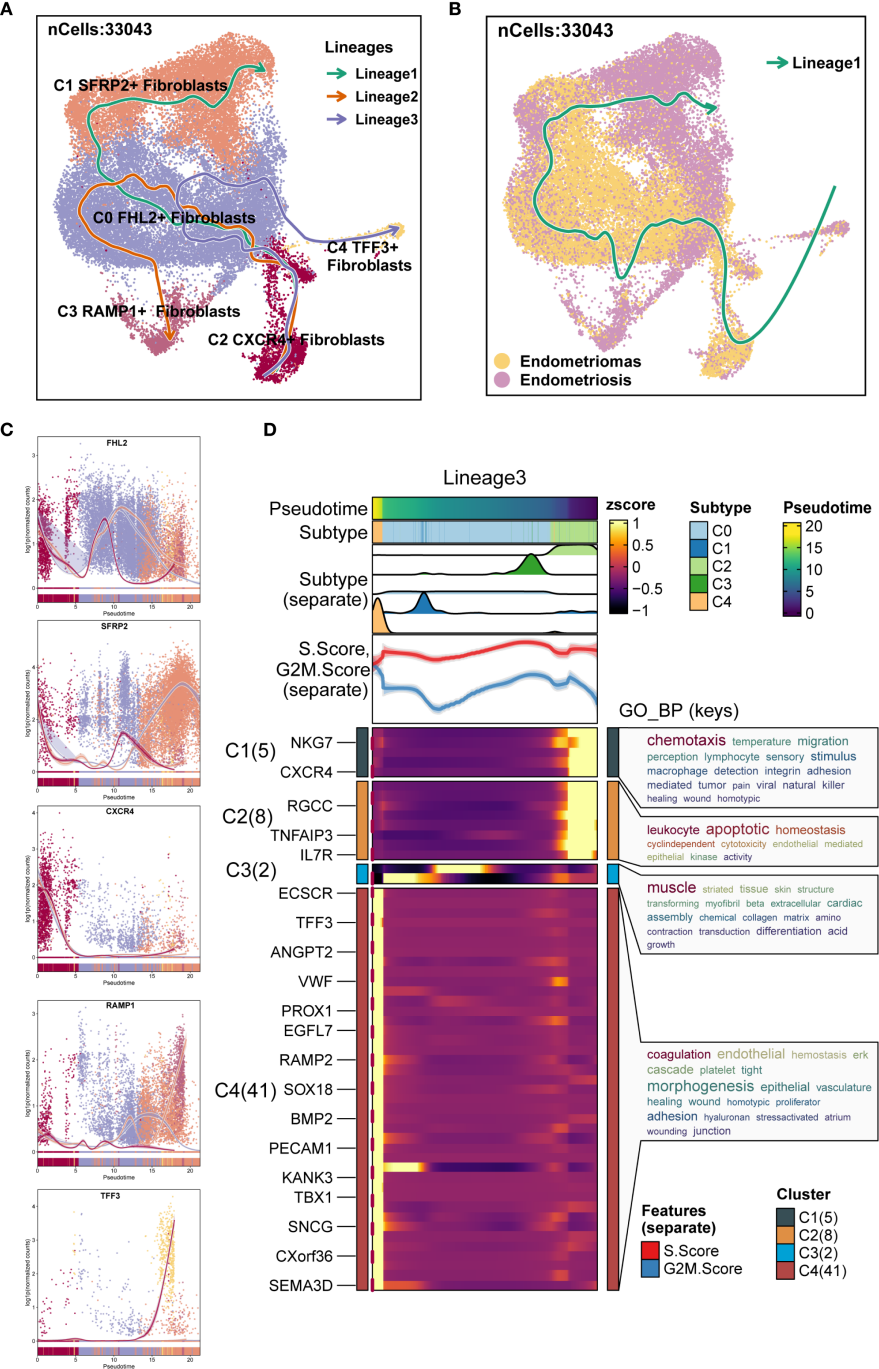
Slingshot analysis of EM Fibroblast subpopulations. **(A)** The Slingshot analysis identified three lineages among the fibroblast subpopulations. Lineage 1: C2 *CXCR4* ^+^ Fibroblast → C0 *FHL2* ^+^ Fibroblast → C1 *SFRP2* ^+^ Fibroblast; Lineage 2: C2 *CXCR4* ^+^ Fibroblast → C0 *FHL2* ^+^ Fibroblast → C3 *RAMP1* ^+^ Fibroblast; Lineage 3: C2 *CXCR4* ^+^ Fibroblast → C0 *FHL2* ^+^ Fibroblast → C4 *TFF3* ^+^ Fibroblast. **(B)** The Slingshot analysis for the two tissue types revealed only one lineage. **(C)** The scatter plots showed the variation of marker genes for each fibroblast subpopulation across the three Slingshot trajectories. **(D)** GOBP enrichment analysis was performed on the DEGs of Lineage 3, and the enriched terms were displayed.

Additionally, Slingshot analysis was employed to investigate the developmental trajectories of EM fibroblast subpopulations. Three lineages were generated, all starting from the C2 *CXCR4* ^+^ Fibroblast subpopulation. The terminal ends of Lineage 1, Lineage 2, and Lineage 3 corresponded to the C1 *SFRP2* ^+^ Fibroblast, C3 *RAMP1* ^+^ Fibroblast, and C4 *TFF3* ^+^ Fibroblast subpopulations, respectively (**Figure 6A**). Based on the Monocle 2 analysis results, we hypothesized that the EM fibroblast subpopulations differentiated from the C2 *CXCR4* ^+^ Fibroblast. Notably, the C2 *CXCR4* ^+^ Fibroblast subpopulation exhibited the highest Cell Stemness Score, along with elevated CNV and G2M scores compared to the other fibroblast subpopulations, piquing our interest in further investigating the C2 *CXCR4* ^+^ Fibroblast subpopulation.

Slingshot analysis of the two tissue types was shown in **Figure 6B**, with results indicating a tendency for Endometriomas to transition towards Endometriosis. The naming genes of the EM fibroblast subpopulations were analyzed across the three lineages, as depicted in **Figure 6C**. The results revealed that the expression of the *CXCR4* gene in the C2 subpopulation was highest at the early stages of the trajectory, gradually decreasing as pseudotime progressed. DEGs enrichment analysis for Lineage 3 was shown in **Figure 6D**. Enrichment analysis of DEGs in the Lineage 3 trajectory revealed that the C2 *CXCR4* ^+^ Fibroblast subpopulation was closely associated with biological processes such as muscle striated tissue formation, skin structure, transforming myofibril, beta extracellular, and transduction differentiation. These findings highlight the potential roles of the C2 subpopulation in tissue repair, extracellular matrix remodeling, and other functions.

### 3.6 Identification of TF-regulatory submodules in EM fibroblast subpopulations

**Figure 7:**
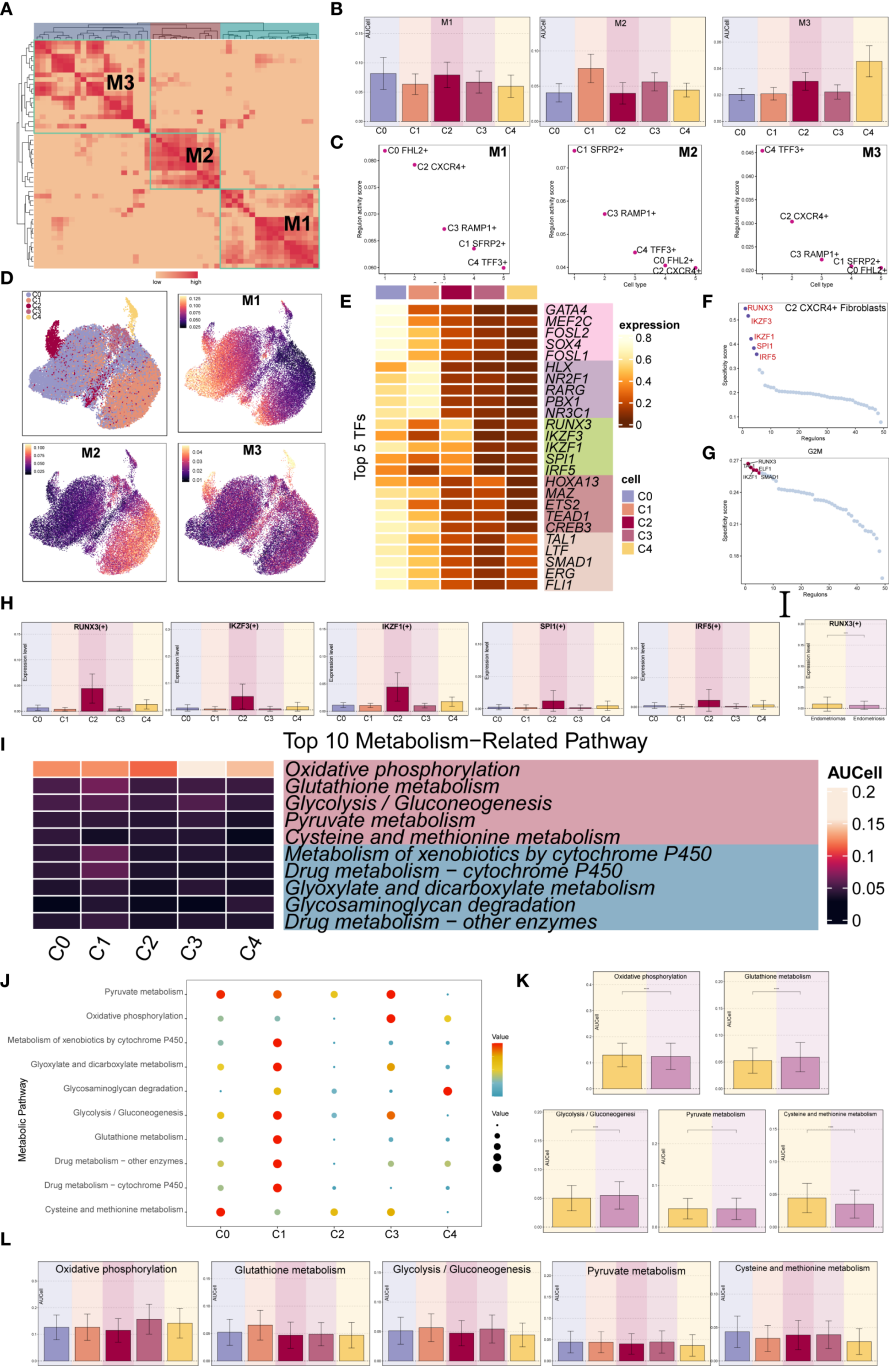
Gene regulatory network and metabolic analysis of EM Fibroblast subpopulations. **(A)** The heatmap displayed the three regulatory modules (M1, M2, M3) across the EM fibroblast subpopulations. **(B)** The bar plots illustrated the expression levels of each fibroblast subpopulation in different regulatory modules. **(C)** The Regulon activity score (RAS) was used to identify the specific relationships between regulons and each cell type. **(D)** The UMAP plots showed the distribution of EM fibroblast subpopulations based on regulatory modules. **(E)** The heatmap presented the top 5 transcription factors (TFs) for the five EM Fibroblast subpopulations. **(F)** The ranking of TFs in the C2 *CXCR4*⁺ Fibroblast subpopulation was shown based on their Regulon specificity score (RSS). (G) The ranking of TFs in G2M was shown based on their RSS. **(H)** The bar plots displayed the expression levels of the top 5 TFs in different Fibroblast subpopulations for C2 *CXCR4* ^+^ Fibroblast. **(I)** The heatmap showed the AUCell values of the top 10 metabolism-related pathways across different EM Fibroblast subpopulations. **(J)** The bubble plot illustrated the scores of the top 10 metabolism-related pathways in different EM Fibroblast subpopulations. **(K)** The AUCell values of the top 5 metabolism-related pathways across different tissue types were shown. **(L)** The AUCell values of the top 5 metabolism-related pathways in different EM Fibroblast subpopulations were displayed. (*P < 0.05;****P < 0.0001).

We used the specific index (CSI) matrix to identify three regulatory modules in the EM fibroblast subpopulations, namely M1, M2, and M3 (**Figure 7A**). The expression levels of TFs in each regulatory module across the fibroblast subpopulations were shown in **Figure 7B**. Additionally, we calculated the Regulon activity score (RAS) to define the specific correspondence between Regulons and each fibroblast subpopulation. **Figure 7C** showed the RAS of each Fibroblast subpopulation across regulatory modules. The results indicated that TFs in the M1 module mainly regulated the C0 *FHL2* ^+^ and C2 *CXCR4* ^+^ Fibroblast. The distribution of the EM Fibroblast subpopulations based on the regulatory modules was shown in **Figure 7D**.

To identify the core TFs of the EM Fibroblast subpopulations, we used pySCENIC to analyze the gene regulatory networks (**Figure 7E**). The results revealed that the top five TFs in the C2 *CXCR4* ^+^ Fibroblast subpopulation were RUNX3, IKZF3, IKZF1, SPI1, and IRF5. **Figure 7H** showed the expression of key TFs across subpopulations. RUNX3, the leading TF in C2 *CXCR4* ^+^ Fibroblast, was linked to EM malignant transformation through hypermethylation (52). Additional analysis found RUNX3 had the highest specificity in the G2M phase and exhibited elevated expression in Endometriomas compared to Endometriosis (**Figures 7F–H**), indicating its potential as a therapeutic target.

We further examined metabolic pathways in fibroblast subpopulations and identified key processes such as oxidative phosphorylation, glutathione metabolism, glycolysis/gluconeogenesis, pyruvate metabolism, and cysteine and methionine metabolism (**Figure 7I**). Their expression levels across subpopulations appeared in **Figures 7J, L**. Glutathione metabolism and glycolysis/gluconeogenesis showed higher activity in Endometriosis tissues, whereas cysteine and methionine metabolism were increased in Endometriomas (**Figure 7K**).

### 3.7 Functional validation of CXCR4 in endometrium-associated cells

**Figure 8:**
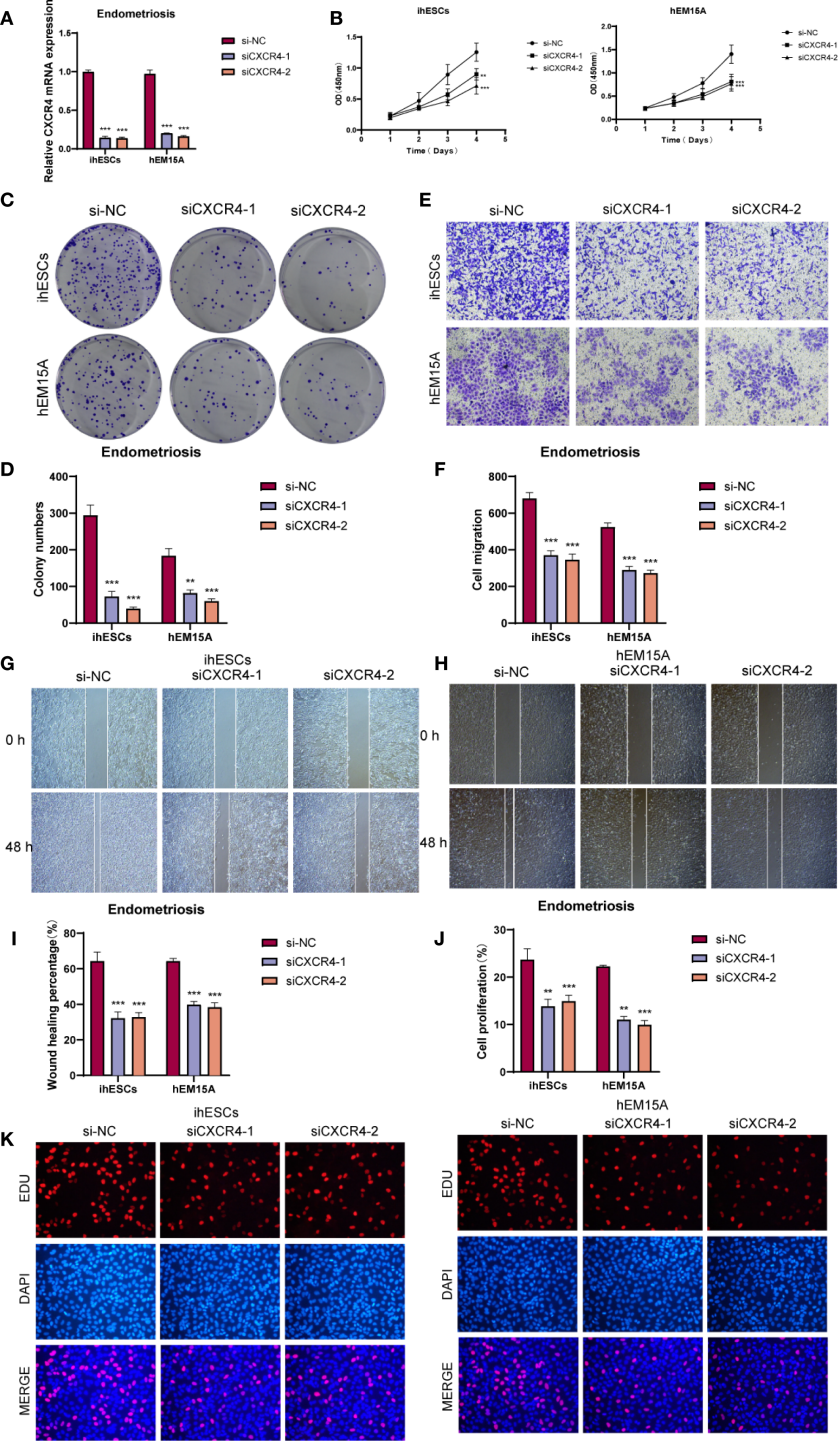
*In vitro* functional assays validated the role of CXCR4. **(A)** qRT-PCR analysis showed that CXCR4 knockdown significantly reduced mRNA expression in ihESCs and hEM15A cells. **(B)** CCK-8 assay indicated that CXCR4 knockdown markedly suppressed the proliferative capacity of both cell lines. **(C, D)** Colony formation assays revealed a significant reduction in clonogenic ability upon CXCR4 knockdown. **(E, F)** Transwell migration assays demonstrated that the number of migrated cells was markedly decreased following CXCR4 knockdown, indicating impaired migratory ability. **(G–I)** Wound healing assays showed that cells with CXCR4 knockdown exhibited slower wound closure compared with controls. **(J, K)** EdU incorporation assays and quantitative analysis further confirmed the inhibitory effect of CXCR4 knockdown on cell proliferation in ihESCs and hEM15A cells. (**P < 0.01; ***P < 0.001).

We investigated the biological role of *CXCR4* in endometrium-associated cells by knocking down its expression in ihESCs and hEM15A cell lines and performing *in vitro* functional assays. qRT-PCR analysis confirmed that *CXCR4* mRNA levels were effectively reduced in the siRNA group compared to controls (**Figure 8A**). The CCK-8 assay suggested that CXCR4 knockdown impaired cell proliferative capacity (**Figure 8B**), and colony formation assays showed a decrease in clonogenic ability in both cell lines (**Figures 8C, D**). To evaluate cell migration, Transwell assays demonstrated a reduction in migrated cell numbers following CXCR4 knockdown (**Figures 8E, F**), and wound healing assays indicated slower wound closure at 48h in CXCR4 knockdown cells compared with controls (**Figures 8G–I**). EdU incorporation assays further suggested a reduction in the proportion of EdU-positive cells after CXCR4 knockdown, indicating a potential inhibitory effect on proliferation (**Figures 8J, K**).

### 3.8 Complex crosstalk network analysis of EM fibroblast subpopulations

**Figure 9:**
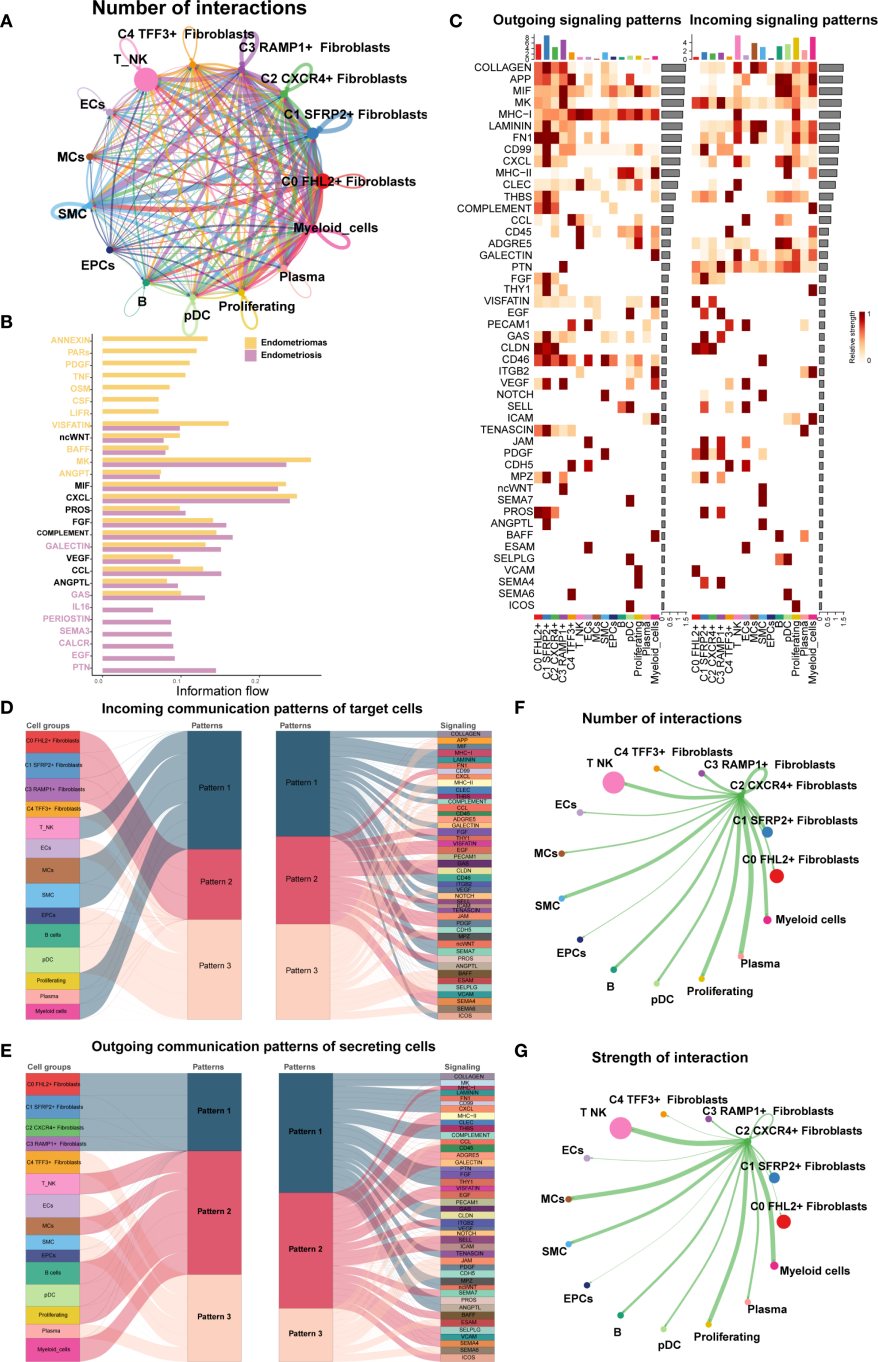
Cell crosstalk network across different cell types in EM. **(A)** The circle plot illustrated the number of cell-cell interactions among all cell types in EM. **(B)** The signal network differences between Endometriomas and Endometriosis were ranked, with the top yellow signal pathways being more enriched in Endometriomas and the bottom red pathways more enriched in Endometriosis. **(C)** The heatmap provided an overview of outgoing and incoming signaling across all cell types. **(D, E)** The alluvial plots displayed the incoming communication patterns of target cells and outgoing communication patterns of secreting cells among all EM cell types. **(F)** The circle plot showed the number of cell interactions with C2 *CXCR4* ^+^ Fibroblast as the source. **(G)** The circle plot illustrated the interaction strengths of C2 *CXCR4* ^+^ Fibroblast as the source of cell crosstalk.

Finally, we used CellChat to analyze the cell-cell communication networks between the EM Fibroblast subpopulations and other cell types. A comprehensive interaction map of all cell types was shown in **Figure 9A**. The results revealed that C2 CXCR4^+^ Fibroblast had particularly strong interactions with SMCs. Differential signaling networks between Endometriomas and Endometriosis suggested that EGF may be more enriched in Endometriosis, while VEGF was similarly enriched in both tissue types (**Figure 9B**).

**Figure 9C** presented an overview of outgoing and incoming signaling across all EM cell types. Notably, C2 CXCR4^+^ Fibroblast played a key role in FN1, CLDN, and CD46 signaling pathways. The incoming signaling pathways in EM Fibroblast subpopulations were primarily associated with Pattern 2, involving pathways such as CD99, FGF, and EGF (**Figure 9D**). In contrast, the outgoing signaling pathways were primarily linked to Pattern 1, including FN1, CD99, and ncWNT pathways (**Figure 9E**).

When C2 CXCR4^+^ Fibroblast were considered as the signal source, the analysis of interaction frequency (**Figure 9F**) and interaction strength (**Figure 9G**) revealed that this subpopulation had stronger and more frequent interactions with T NK cells and SMCs.

### 3.9 FN1 signaling pathway network analysis

**Figure 10:**
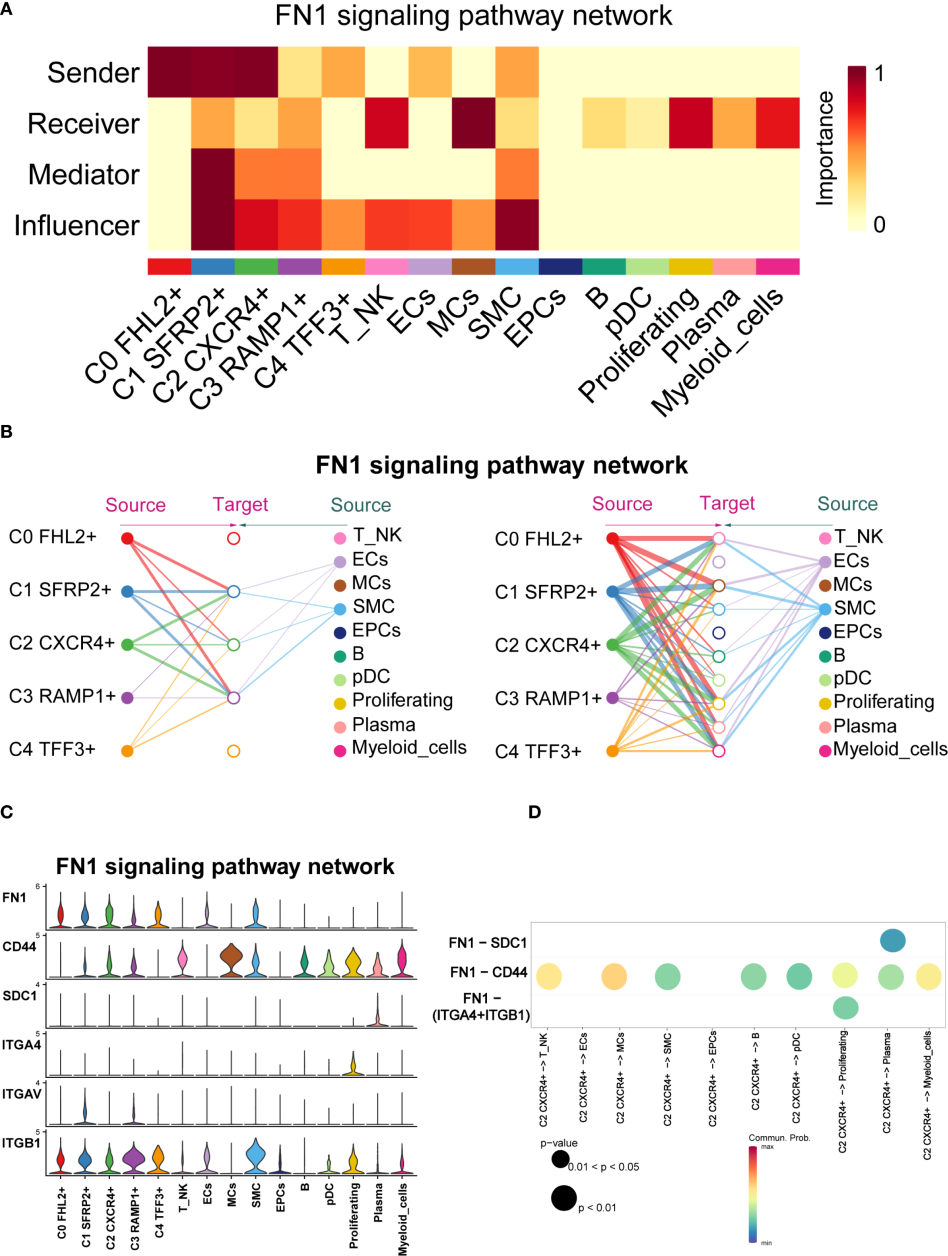
FN1 signaling pathway network across cell types in EM. **(A)** The heatmap displayed the four centrality scores of FN1, highlighting the relative importance of each cell type as a sender, receiver, mediator, and influencer. **(B)** The hierarchical plot illustrated the intercellular communication network of FN1 signaling. The left and right sides respectively displayed the autocrine and paracrine signals toward fibroblasts and other cell types. Solid and open circles represented source and target cell types, with circle sizes proportional to the number of cells in each type. **(C)** The violin plot showed the expression distribution of six genes involved in the inferred FN1 signaling pathway network. **(D)** The bubble plot compared the key ligands and receptors involved in the FN1 signaling pathway network between the C2 *CXCR4* ^+^ Fibroblast subpopulation and other subpopulations.

Previous studies have highlighted the close association between FN1 and EM (53). To further explore FN1 signaling in EM, we visually analyzed the pathway and used centrality scores to assess the importance of various cell types within the FN1 network. The results revealed that fibroblast subpopulations exhibited higher centrality scores compared to other cell types. Specifically, the C1 *SFRP2* ^+^ Fibroblast subpopulation scored highly as Sender, Mediator, and Influencer, while the C2 *CXCR4* ^+^ Fibroblast subpopulation scored highly as Sender and Influencer (**Figure 10A**).

CellChat analysis indicated that the fibroblast subpopulations were the primary sources of FN1, signaling through autocrine and paracrine mechanisms (**Figure 10B**). **Figure 10C** showed the expression distributions of six genes in the FN1 signaling pathway. FN1, CD44, and ITGB1 were highly expressed in multiple cell types. **Figure 10D** illustrated the regulatory effects of the C2 *CXCR4* ^+^ Fibroblast subpopulation on various cell types within this pathway.

CellChat inferred that the FN1-CD44 ligand-receptor interaction plays a crucial role in communication between C2 *CXCR4* ^+^ Fibroblast and other cell types (T NK, MCs, SMC, B, pDCs, Proliferating, Plasma, and Myeloid cells). Significant ligand-receptor interactions between the C2 *CXCR4* ^+^ Fibroblast subpopulation (as the signal sender) and various recipient cell types were shown in **Supplementary Figure 1A**.

### 3.10 Spatial transcriptomic analysis of endometriotic lesions

**Figure 11:**
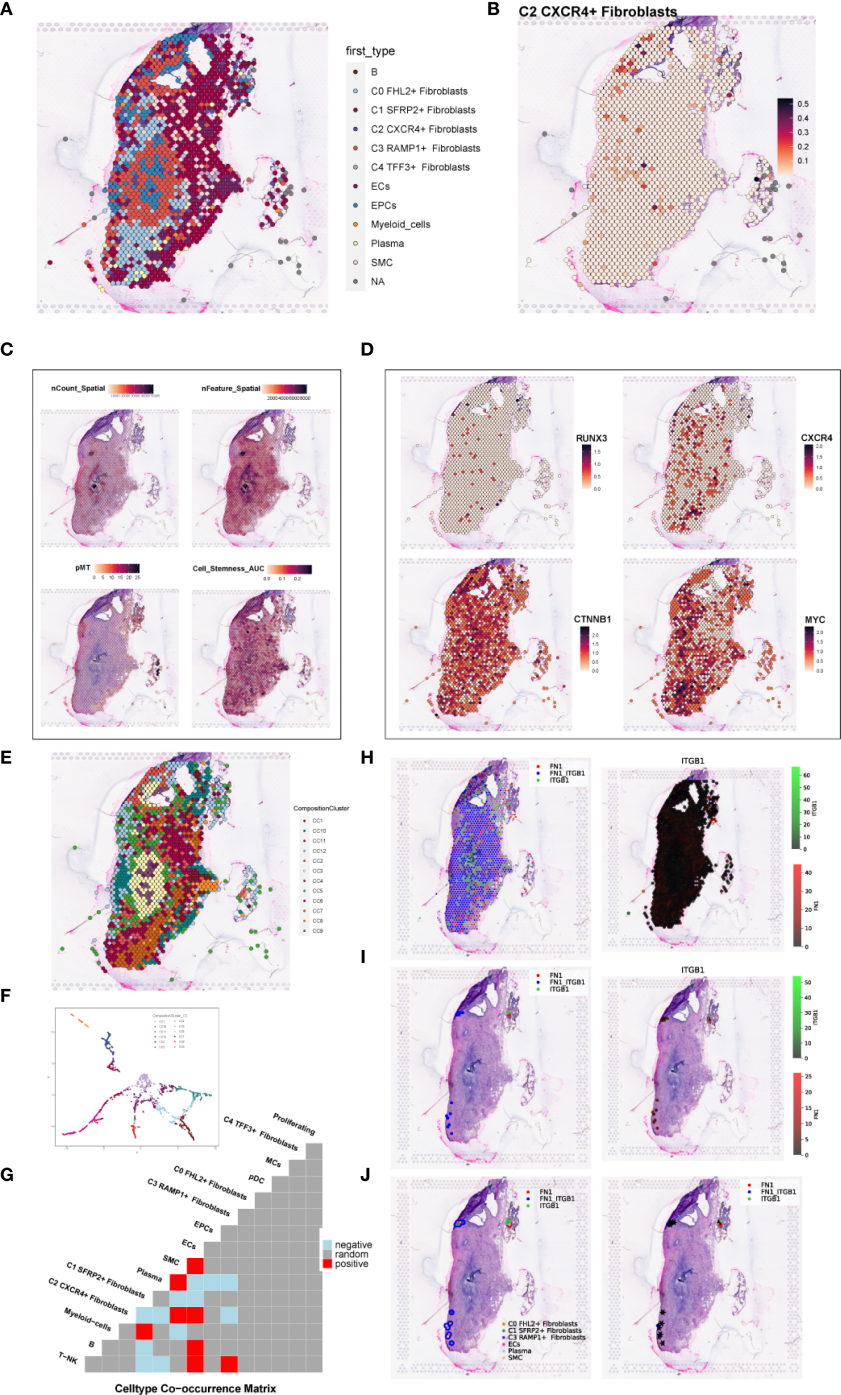
Spatial transcriptomic features of an endometriosis lesion from patient GSM6690475. **(A)** ST map showing the dominant cell type (first_type) per spot as identified by RCTD. **(B)** ST map showing the relative abundance of C2 *CXCR4*+ fibroblast, with a color gradient from light red (low abundance, 0.1) to deep red (high abundance, 0.5). **(C)** Spatial feature maps displaying nCount_Spatial, nFeature_Spatial, mitochondrial gene percentage (pMT), and cell stemness AUC across the tissue section. **(D)** ST maps showing the spatial expression patterns of the top TF RUNX3, the C2 fibroblast marker gene *CXCR4*, and stemness-related genes *CTNNB1* and *MYC*. **(E)** ST map displaying the spatial distribution of the 12 clusters across the tissue section based on ISCHIA clustering. **(F)** UMAP plot illustrating the transcriptomic similarity and separation among the 12 spatial clusters. **(G)** Co-occurrence heatmap of spatial clusters. Red indicated positive co-occurrence, blue indicated negative co-occurrence, and grey indicated no significant association. **(H)** Binary coexpression map of FN1–ITGB1 across all spots, indicating global ligand–receptor interaction distribution. **(I)** Refined coexpression plots within significant spots: binary (left) and continuous (right) representations highlight regions with strong interactions. **(J)** Spatial visualization with spot annotations showing that FN1–ITGB1 signaling was predominantly localized at the tissue periphery.

To investigate the spatial distribution of cell types in endometriotic lesions, we analyzed spatial transcriptomic data from tissue sections GSM6690475 and GSM6690476. We first applied Robust Cell Type Decomposition (RCTD) to deconvolute the cellular composition of GSM6690475 and visualized the dominant cell type (first_type) for each spatial spot using an ST map (**Figure 11A**). A separate ST map revealed the spatial abundance of C2 *CXCR4*+ Fibroblast, showing their restricted localization (**Figure 11B**). Spatial feature maps further illustrated key quality control and biological indicators, including nCount Spatial, nFeature Spatial, pMT, and stemness AUC (**Figure 11C**). The spatial expression of representative transcription factors and marker genes, including RUNX3, CXCR4, CTNNB1, and MYC, was also visualized, confirming their localized enrichment patterns (**Figure 11D**).

To explore higher-order spatial structure, we performed unsupervised clustering using ISCHIA, which grouped all spatial spots into 12 distinct clusters. These clusters were visualized both in spatial context by ST map (**Figure 11E**) and in reduced dimensional space by UMAP plot (**Figure 11F**). A co-occurrence heatmap revealed the spatial relationships among clusters, indicating that C2 *CXCR4*+ Fibroblast strongly co-occurred with SMCs and ECs, while showing negative co-occurrence with plasma cells (**Figure 11G**). These findings aligned with previous CellChat-based interaction results, suggesting a spatially coordinated immune–stromal microenvironment.

We further employed the stLearn Python package to analyze ligand–receptor interactions within the spatial context. Focusing on the FN1–ITGB1 ligand–receptor pair, we first performed global screening using binary coexpression analysis across all spatial spots to assess overall signaling intensity (**Figure 11H**). We then refined the analysis to high-confidence regions by visualizing both binary and continuous coexpression in statistically significant spots (**Figure 11I**). Lastly, spot-level annotations were added to visualize the spatial pattern of FN1–ITGB1 signaling (**Figure 11J**). The results demonstrated that this interaction was predominantly concentrated at the tissue periphery, suggesting a localized stromal–integrin signaling axis with potential roles in boundary formation or cell–matrix remodeling.

**Figure 12:**
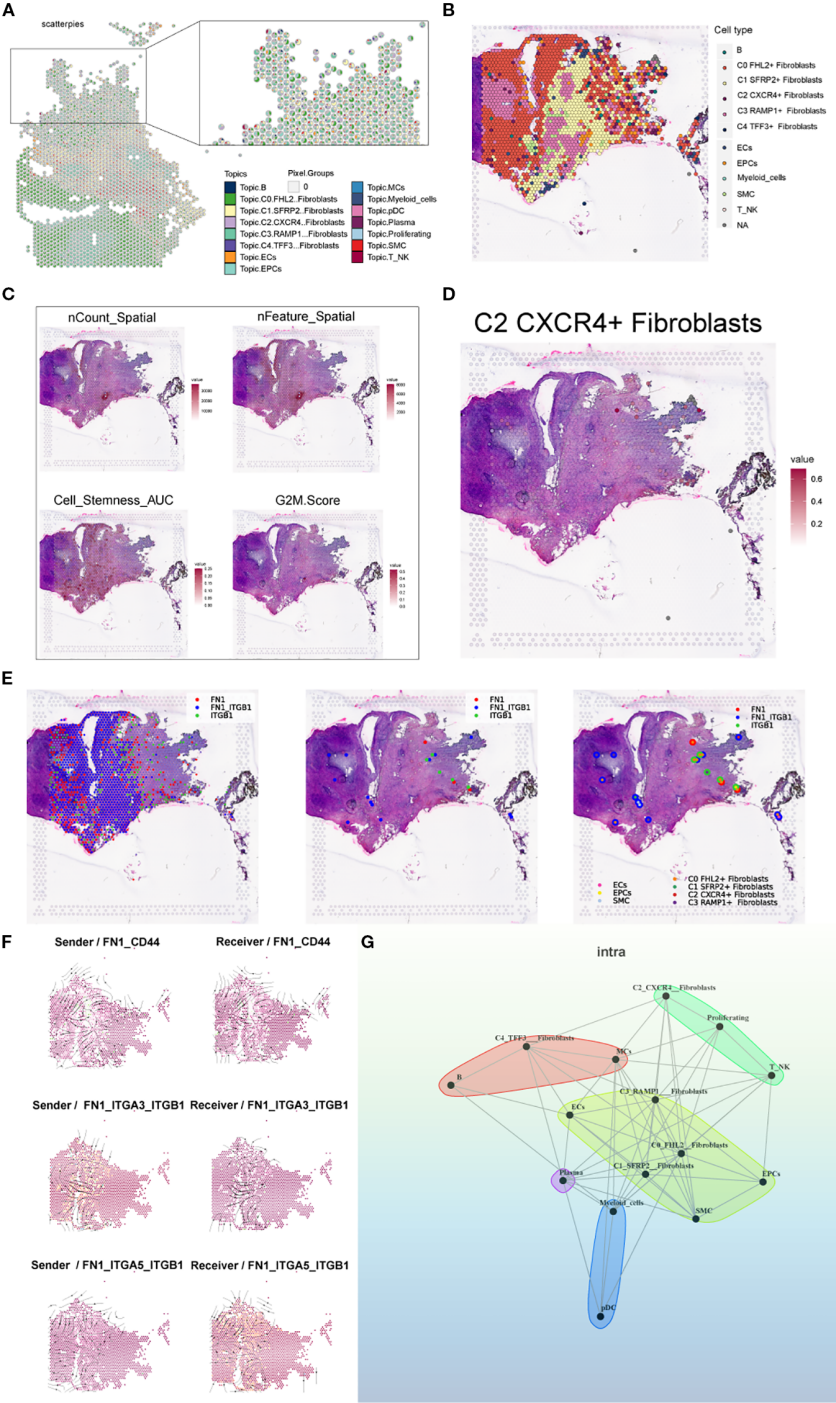
Spatial transcriptomic features of an endometriosis lesion from patient GSM6690476. **(A)** Pie charts showing the proportion of each cell type at individual spatial spots based on RCTD results. **(B)** Spatial map displaying the dominant cell type (first_type) at each spot based on RCTD deconvolution results. **(C)** ST maps showing the distribution and intensity of nCount_Spatial, nFeature_Spatial, cell stemness AUC, and G2M Score across the tissue. **(D)** Spatial distribution map showing the C2 *CXCR4* ^+^ Fibroblast subpopulation. **(E)** Binary LR coexpression plots of FN1 and ITGB1 from stlearn: all spots, significant spots, and with spot annotations. **(F)** Spatial distribution of sender (left) and receiver (right) cells for three key FN1-mediated interactions: FN1–CD44, FN1–ITGA3–ITGB1, and FN1–ITGA5–ITGB1. **(G)** Cell–cell interaction network inferred by the MISTyR intraview model (spatial scale, importance ≥ 0.2), with nodes representing RCTD-identified cell types and edge thickness denoting interaction strength.

To further validate and compare the spatial transcriptomic architecture of endometriotic lesions, we analyzed tissue section GSM6690476. Pie charts based on RCTD deconvolution showed the proportional distribution of cell types at each spatial spot, and a spatial map highlighted the dominant cell type across the tissue (**Figures 12A, B**). Spatial feature plots revealed gradients in sequencing quality and biological properties, including nCount Spatial, nFeature Spatial, stemness AUC, and G2M score, indicating spatial heterogeneity in proliferative and stem-like properties (**Figure 12C**). The C2 *CXCR4* ^+^ Fibroblast subpopulation showed distinct spatial localization, reinforcing its relevance to lesion architecture (**Figure 12D**).

Using stLearn, we visualized the spatial coexpression of FN1 and ITGB1 across all spots, significant spots, and annotated regions, revealing spatially confined ligand–receptor activity (**Figure 12E**). We further mapped the spatial distribution of sender and receiver cells for three key FN1-mediated interactions—FN1–CD44, FN1–ITGA3–ITGB1, and FN1–ITGA5–ITGB1—highlighting interaction hotspots enriched at the stromal–epithelial interface (**Figure 12F**). Finally, a MISTyR-inferred cell–cell interaction network at the intratissue proximity level revealed strong context-dependent communications, particularly involving C2 Fibroblast, suggesting their central role in coordinating the local cellular microenvironment (**Figure 12G**).

## 4 Discussion

This study conducted an in-depth molecular characterization of EM-associated fibroblast subpopulations using scRNA-seq, revealing the heterogeneity of different cell subpopulations and their potential roles in EM progression. Through comprehensive analysis of scRNA-seq data from EM patients, we confirmed the complex cellular composition of the EM microenvironment, with particular emphasis on the abundance of immune cells and fibroblast subpopulations. Among the 35 identified cell clusters, T NK cells and Fibroblast represented the dominant subpopulations, further highlighting the close relationship between immune responses and the onset and progression of EM.

Fibroblast subpopulations in EM exhibited significant heterogeneity, and these fibroblast subpopulations were classified into five subpopulations: C0 *FHL2* ^+^, C1 *SFRP2* ^+^, C2 *CXCR4* ^+^, C3 *RAMP1* ^+^, and C4 *TFF3* ^+^. Notably, C2 *CXCR4* ^+^ Fibroblast subpopulation exhibited the highest Cell Stemness Score, along with elevated CNV and G2M Scores, suggesting that this subpopulation may be in a more active proliferative state and could serve as the initiating subpopulation for fibroblast differentiation. Moreover, Pseudotime analysis revealed the developmental trajectory of the fibroblast subpopulations, indicating that C2 *CXCR4* ^+^ Fibroblast may act as precursor cells for the other subpopulation, further highlighting their key role in the fibrotic progression of EM. Additionally, the TF regulatory network analysis of Fibroblast subpopulations identified RUNX3 as one of the core TFs in C2 *CXCR4* ^+^ Fibroblast. RUNX3 was found to be closely associated with the malignant transformation of EM (52, 54), with its expression level in Endometriomas being significantly higher than in Endometriosis, emphasizing its potential as a therapeutic target for EM.

FN1, a glycoprotein in the extracellular matrix, is involved in key cellular processes, including cell adhesion, migration, wound healing, coagulation, and even metastasis (55). Previous systematic reviews and meta-analyses have suggested that the FN1 gene variant rs1250248 may influence the development of EM (56). Furthermore, the significant role of FN1 in EM has been corroborated by studies such as that of Pagliardini et al. (57). Beliard et al. proposed that FN1 may contribute to the persistence of endometriotic lesions and found increased FN1 receptor expression in the endometriotic glands of the peritoneal lesions compared to the eutopic endometrium from healthy controls (58). Recent research has indicated that EM-associated mesothelial cells interact with ectopic endometrial stromal cells through the FN1-integrin complex and suggested that these mesothelial cells may induce progesterone resistance in ectopic stromal cells via the FN1-PI3K-AKT pathway (59). However, the potential relationship between FN1 and Fibroblast remains insufficiently explored.

This study found that FN1 was highly expressed in fibroblast subpopulations associated with EM, particularly in the C1 *SFRP2* ^+^ Fibroblast and C2 *CXCR4* ^+^ Fibroblast subpopulation. Furthermore, our research suggested that the FN1 signaling pathway may play a key role in the fibrosis process and immune microenvironment shaping in EM by regulating intercellular communication among Fibroblast. CellChat analysis showed that C1 *SFRP2* ^+^ and C2 *CXCR4* ^+^ Fibroblast were main sources of FN1 and likely shaped the EM microenvironment via key ligand-receptor pairs like FN1-CD44, impacting immune and stromal cells. The strong links between C2 *CXCR4* ^+^ Fibroblast, T NK cells, and SMCs suggest this group may play a role in immune regulation or evasion in EM. Further analysis indicated that FN1-ITGB1 and FN1-CD44 signaling axes exhibited high interaction frequencies in the EM microenvironment, implying that FN1 may play an essential role in the fibrosis process of EM by mediating adhesion and signal transduction between Fibroblast, immune cells, and smooth muscle cells. The varying FN1 signaling activity across fibroblast subpopulations indicated they had different roles in EM pathology. C1 *SFRP2* ^+^ Fibroblast likely contributed to EM remodeling, while C2 *CXCR4* ^+^ Fibroblast were more involved in ongoing inflammation and immune regulation. Spatial transcriptomics confirmed that C2 *CXCR4* ^+^ Fibroblast were enriched in specific tissue areas, with FN1 signaling—especially via FN1–ITGB1—localized to the tissue edge and stromal–epithelial border. Combined analyses with RCTD, stLearn, ISCHIA, and MISTyR revealed that C2 *CXCR4* ^+^ Fibroblast functioned as spatial hubs coordinating fibrosis and immune responses. These findings highlighted FN1 signaling as a crucial spatial and functional factor in EM progression.

Our functional assays demonstrated that CXCR4 knockdown impaired proliferation, clonogenicity, and migration in ihESC and hEM15A cells. These results support the biological relevance of our single-cell and spatial findings, but further validation in primary tissues and *in vivo* animal models is still required.

We acknowledge that the number of samples included for both single-cell and spatial transcriptomics analyses is limited. While this may constrain the generalizability of our findings and underestimate inter-patient variability, the observed patterns were consistent across multiple patients and analyses. Future studies with larger cohorts will be critical to validate and extend these findings. Based on previous studies and our comprehensive multi-omics analysis, we propose that FN1 plays a key role in the fibrotic process of endometriosis, which regulates the inflammatory response through key fiber cell subsets and through interaction with immune cells. Our findings provide a rationale for clinical translation, as FN1 plays a pivotal role in fibroblast-mediated fibrosis and immune remodeling. Targeting FN1 interactions with receptors such as ITGB1 or CD44 may constitute a promising non-hormonal therapeutic strategy, warranting further preclinical evaluation and potential clinical development.

## 5 Conclusion

This study employed an integrated multi-omics approach combining scRNA-seq and spatial transcriptomics to characterize the heterogeneity and functional diversity of fibroblast subpopulations in endometriosis. We identified FN1 signaling, particularly within the C2 *CXCR4* ^+^ Fibroblast subpopulation, as a central mediator of fibrosis and immune remodeling in ectopic lesions. These findings enhance our understanding of the cellular and molecular mechanisms driving EM progression and highlight FN1 as a potential target. Further studies are warranted to elucidate the complex fibroblast-immune interactions and to validate FN1-targeted interventions for improved management of endometriosis.

## Data Availability

The original contributions presented in the study are included in the article/**Supplementary Material**. Further inquiries can be directed to the corresponding author.

## Supplementary material

Supplementary Figure 1 Cellular communication between C2 *CXCR4* ^+^ Fibroblast and other clusters. **(A)** The bubble plot compared the key ligands and receptors involved in signaling between C2 *CXCR4* ^+^ Fibroblast (as the source) and other cell types.
