# Supplementary figures and images for: Fibroblast heterogeneity and FN1-mediated signaling in endometriosis revealed by single-cell and spatial transcriptomics
Source: Front Immunol. 2025 Oct 13;16:1680849. doi: 10.3389/fimmu.2025.1680849 (PMC12554657; doi:10.3389/fimmu.2025.1680849)

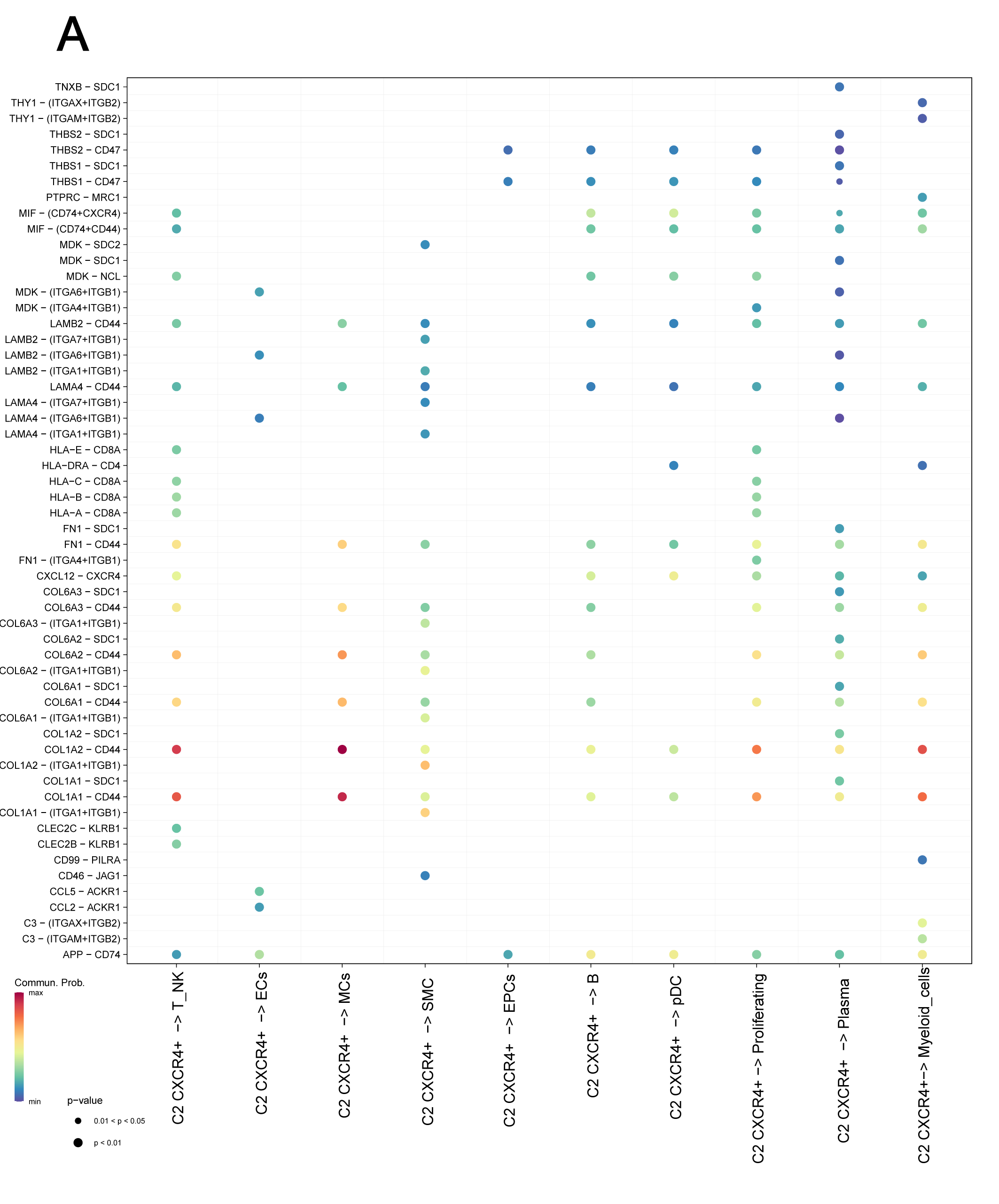

Supplement: Supplementary Figure 1 — Cellular communication between C2 CXCR4 + Fibroblast and other clusters. (A) The bubble plot compared the key ligands and receptors involved in signaling between C2 CXCR4 + Fibroblast (as the source) and other cell types. [file Image1.tif]
